# Calcineurin inhibitor–induced pain syndrome: an uncommon side effect of immunosuppressive therapy after allogeneic hematopoietic stem cell transplant. a case report and review of the literature

**DOI:** 10.1007/s00277-026-07079-w

**Published:** 2026-05-27

**Authors:** Rachele De Domenico, Gabriele Magliano, Enrico Morello, Silvia Zanon, Mirko Farina, Vera Radici, Domenico Russo, Michele Malagola, Daniele Avenoso

**Affiliations:** Unit of Blood Diseases and Bone Marrow Transplantation, Department of Clinical and Experimental Science, University of Brescia, ASST Spedali Civili di Brescia, Brescia, Italy

**Keywords:** CIPS, Pain, AML, Tacrolimus, Ciclosporin, GVHD, Calcineurin inhibitor-induced pain syndrome

## Abstract

Calcineurin inhibitor–induced pain syndrome (CIPS) is a rare complication of immunosuppressive therapy, predominantly described in solid organ transplantation and only occasionally after allogeneic hematopoietic stem cell transplantation (allo-HSCT). We report an atypical early presentation of CIPS in a 57-year-old woman with adverse-risk acute myeloid leukemia undergoing matched sibling donor allo-HSCT. GVHD prophylaxis included ciclosporin, methotrexate, and antithymocyte globulin. On day + 2, the patient developed severe, stabbing back pain refractory to opioids and partially responsive to pregabalin. Extensive cardiologic, neurologic, radiologic, and laboratory workup was negative except for mild alkaline phosphatase elevation; ciclosporin levels were therapeutic. After exclusion of alternative etiologies, CIPS was suspected (Naranjo score 7). Pain resolved rapidly following ciclosporin discontinuation and steroid introduction, with no recurrence during subsequent tacrolimus therapy.

## Introduction

The curative effect of allogeneic hematopoietic stem cell transplant (allo-HSCT) is a balance between the graft-versus-tumor effect and the graft-versus-host disease (GVHD). Current GVHD prophylaxis strategies rely on the combination of antimetabolite or alkylating agents with either mTOR pathway inhibitors (i.e. sirolimus) or calcineurin inhibitors (i.e. ciclosporin and tacrolimus, CNIs), which are associated with multiple side effects. Nephrotoxicity, neurotoxicity, and hypertension are frequently reported [1, 2], however, less common but clinically challenging adverse effects, such as myalgias and arthralgias, have also been described. Calcineurin inhibitor-induced pain syndrome (CIPS) is a rare but recognized complication of CNIs therapy in transplant recipients [3, 4]. Herein, we present the case of an allo-HSCT patient who developed musculoskeletal adverse events likely because of CNI administration.

## Case presentation

In January 2024 a 57-year-old woman was diagnosed with acute myeloid leukemia (AML); the molecular stratification (DNMT3A, SRSF2, RUNX1, IDH2 mutated) allocated the patient into the poor prognosis group of the European Leukaemia Net 2022 classification [5]. After successful first line induction chemotherapy, she underwent allo-HSCT from a matched sibling donor in January 2025, following conditioning with FT10 [6] and low dose total body irradiation (200 cGY) on day − 1. GVHD prophylaxis consisted of ATLG, ciclosporin 3 mg/Kg from day – 1 and short-course methotrexate (15 mg/m² on day + 1, 10 mg/ m² on day + 3 and day + 6). Ciclosporin dose was adjusted to maintain serum levels between 150 and 200 ng/mL. On day + 2, the patient reported severe intermittent, sharp, stabbing-like back pain, occurring both at rest and during movement, especially worsening at night, refractory to multiple classes of analgesics, including paracetamol, oxycodone and fentanyl. Ultimately, the introduction of pregabalin provided moderate symptomatic relief.

Several investigations were performed, including ECG and echocardiography, chest X-ray, full spine X-ray and magnetic resonance imaging (MRI) and whole-body CT scan, all of which were unremarkable. Neurological evaluation was normal. Brain MRI was not performed as the patient did not show any sign or symptom of posterior reversible encephalopathy syndrome (PRESS).

All laboratory tests were within normal limits (including serum calcium and phosphate levels), except for an increased level of alkaline phosphatase (122–142 U/L). Vitamin D levels were not assessed at the time of symptom onset. Ciclosporin level was 144 ng/mL at pain onset. Peripheral blood smear microscopic examination did not reveal any blast cell, ruling out disease relapse as an etiology of the pain. In the absence of an alternative diagnosis, the clinical presentation was suggestive of an uncommon adverse event related to ciclosporin. The Naranjo Adverse Drug Reaction Probability Scale was applied to this patient obtaining a score of 7, indicating that ciclosporin may have been the cause of pain [7]. In a first attempt, ciclosporin was administered by continuous intravenous infusion without clinical benefit. Consequently, ciclosporin was discontinued on day + 20 and the patient was started on methylprednisolone 0.5 mg/kg for GVHD prophylaxis. From the following day, pain completely resolved, with no recurrence after tapering and discontinuation of the analgesics. Oral tacrolimus was initiated alongside the tapering of methylprednisolone. The patient remained pain free from day + 21 till day + 100, when tacrolimus was discontinued. Notably, when she developed chronic GVHD of the skin and the mouth on day + 230, tacrolimus was reintroduced without any pain relapse.

## Discussion

CIPS is an established rare pain syndrome seen in recipients of solid organ transplants (SOT) [3, 4]. Although CIPS is more common among SOT recipients [8, 9], cases are reported even within allo-HSCT patients as summarized in Table 1 [10, 11]. It can affect both adult and pediatric patients [12, 13].

**Table 1:** Review of CIPS cases reported in literature among solid organ and hematopoietic stem cells transplant recipients

Author (year)	Type of transplant (organ or stem cells source)	Type of donor	IST involved	Type of patient	Sex	Symptoms
Current	Allo-HSCT (PSCB)	MSD	CsA	Adult	F	Back pain
Bouteiller et al. (1989) [14]	SOT (kidney, heart)	NS	CsA	Adult	NS	Lower limb pain
Naredo Sánchez et al. (1994) [15]	SOT (kidney)	NS	CsA	Adult	F	Lower limb pain
Villaverde et al. (1999) [16]	SOT (kidney)	DD	FK506	Adult	F	Lower limb pain
Malat et al. (2002) [17]	SOT (liver)	DD	FK506	Pediatric	F	Lower limb pain
Franco et al. (2004) [18]	SOT (kidney) SOT (kidney)	NS NS	FK506 FK506	Adult Adult	F M	Bilateral knee pain Bilateral knee pain
Kida et al. (2004) [19]	Allo-HSCT (UCB) Allo-HSCT (UCB)	MUD MUD	CsA CsA	Adult Adult	M F	Lower limb pain Lower limb pain
Kida et al. (2004) [11]	Allo-HSCT (BM)	MUD	FK506	Adult	M	Lower limb pain
Collini et al. (2006) [20]	SOT (kidney) SOT (kidney)	DD DD	FK506 CsA	Adult Adult	M M	Joints pain Bilateral feet pain
Fujii et al. (2006) [21]	Allo-HSCT (BM)	MMUD	FK506	Adult	F	Lower limb pain
Takashima et al. (2006) [22]	Allo-HSCT (BM)	MUD	FK506	Adult	F	Lower limb pain
Noda et al. (2008) [23]	Allo-HSCT (NS)	NS	FK506	Adult	F	Lower limb pain
Lavoratore et al. (2009) [12]	Allo-HSCT (NS)	NS	CsA	Pediatric	M	Lower limb pain
Nishikawa et al.(2009) [13]	Allo-HSCT (BM)	MSD	Fk506	Pediatric	M	Lower limb pain
Breteinstein et al. (2011) [24]	SOT (kidney)	NS	CsA	Adult	M	Lower limb pain
Kakihana et al. (2012) [10]	Allo-HSCT (UCB, BM, PBSC)	MSD/ MUD/ MMuD	Both CsA and FK506	Adult	Both M and F	Lower limb pain
Prates et al. (2012) [25]	SOT (kidney)	DD	FK506	Pediatric	F	Abdominal pain
Gurin et al. (2012) [26]	SOT (kidney)	LD	FK506	Adult	F	Lower limb pain
Sahay et al. (2013) [27]	SOT (lung)	NS	FK506	Adult	M	Joints pain
Chapin et al. (2013) [28]	SOT (kidney)	DD	FK506	Adult	M	Lower limb pain
Ayyala et al. (2016) [29]	Allo-HSCT (NS)	MSD	FK506	Pediatric	F	Lower limb pain
Taşoğlu et al. (2016) [30]	SOT (liver)	NS	FK506	Adult	M	Arms and legs pain
Watanabe et al. (2017) [31]	Allo-HSCT (NS)	MMUD	FK506	Adult	M	Lower limb pain
Ishida et al. (2017) [32]	SOT (kidney)	LD	FK506	Adult	F	Joints pain
Mohideen et al. (2018) [9]	SOT (liver and kidney)	NS	FK506	Adult	F	Lower limb pain
Udomkarnjanarun et al. (2018) [8]	SOT (kidney)	DD	FK506	Adult	F	Back pain
Wei et al. (2018) [33]	Allo-HSCT (NS)	Haplo	FK506	Adult	M	Hip and lower limb pain
Chadha et al. (2019) [34]	SOT (Heart) SOT (Kidney)	DD DD	FK506 FK506	Adult Adult	M F	Ankles and feet pain Ankles and feet pain
Varma et al. (2020) [35]	Allo-HSCT(NS) Allo-HSCT (NS)	MSD MUD	FK506 FK506	Adult Adult	M F	Lower limb pain Arms and legs pain
Ilonze et al. (2021) [36]	SOT (Heart)	DD	FK506	Adult	M	Hips, lower limb and abdominal pain

SOT = solid organ transplant, allo-HSCT = allogenic hematopoietic stem cell transplant, PSCB = peripheral blood stem cells, BM = bone marrow, UCB = umbilical cord blood, MSD = matched sibling donor, MUD = matched unrelated donor, MMUD = mismatch unrelated donor, Haplo = haploidentical donor, DD = deceased donor, LD = living donor, NS = not stated; ITS = immunosuppressive therapy, CsA = ciclosporin, FK506 = tacrolimus

CIPS diagnosis represents a challenge due to the lack of well-established clinical, laboratory, and radiological criteria [37]. CIPS typically presents with severe, symmetrical, debilitating lower limb pain [13, 20, 38], but clinical features and manifestations may vary, as a case associated with back pain in a kidney transplant recipient has been reported [8]. Physical examination of the affected area is usually unremarkable [4, 10]. Blood CNIs level may be increased or within the normal range at the onset of pain [4]. A common laboratory finding is elevated alkaline phosphatase, as in our case [8, 35, 39].

Typical radiographic features include bone marrow edema on MRI and increased uptake on bone scintigraphy, whereas X- ray is usually unremarkable [34, 40]. However the absence of suggestive imaging findings do not exclude CIPS. Indeed, MRI sensitivity appears variable, and several cases with clinically consistent CIPS have been reported without evidence of bone marrow edema. Therefore, imaging should be interpreted as supportive rather than diagnostic, and clinical judgment remains central [23, 25]. The diagnosis of CIPS is one of exclusion as more common causes of musculoskeletal, vascular, ischemic and neuropathic pain must first be ruled out. In our case, the strong temporal association between ciclosporin administration and symptom onset, the lack of response to analgesics, and the rapid resolution after drug discontinuation further reinforce the causal relationship, despite the absence of radiological confirmation.

While some of the radiologic features of CIPS are also shared by osteonecrosis and reflex sympathetic dystrophy syndrome (RSDS), these latter post-transplant bone pain syndromes differ significantly in clinical course. Osteonecrosis pain is permanent, weight-dependent and usually localized to the hips [41]. RSDS is distinguished clinically from CIPS by its asymmetric pain distribution, its upper limb predominance and its characteristic association with extremity edema, vasomotor instability and trophic skin changes [41]. The pathophysiology of CIPS is still unknown: in vivo animal models, CNIs induce high bone turnover, as evidenced by elevated alkaline phosphatase levels during therapy [42], but also reduce osteoblastogenesis, thereby impairing bone metabolism with an increased risk of bone fragility and fractures. In addition, CNIs cause intraosseous vasoconstriction and hypoperfusion, leading to bone marrow edema and compartment syndrome [43–45].

Furthermore, CNIs affect nociceptive processes by activating the nitric oxide pathway and modulating gamma-aminobutyric acid (GABA) and N-methyl-D-aspartate (NMDA) receptors, ultimately enhancing neural excitability and pain sensitivity [37, 46–49].

The management of CIPS remains controversial: current strategies include dose reduction of CNIs, gradual replacement with alternative immunosuppressive agents and the use of gabapentinoids (i.e. gabapentin, pregabalin) to address neuropathic pain mechanisms [30, 31]. Calcium channel blocker [4, 50, 51] and bisphosphonates [52] might also be beneficial. Interestingly, in our case, tacrolimus did not reproduce symptoms despite belonging to the same pharmacological class. This observation may reflect pharmacokinetic and pharmacodynamic differences between calcineurin inhibitors, including variability in blood-brain barrier penetration, neurotoxicity profiles, and individual susceptibility. Moreover, interpatient variability in drug metabolism and target sensitivity may contribute to differential nociceptive effects. Similar clinical scenarios have been described, where switching between calcineurin inhibitors resulted in symptom resolution without recurrence, supporting the safety of this strategy. Our report highlights an uncommon manifestation of a rare drug adverse reaction. To our knowledge this is the first reported case of potential CIPS in an allo – HSCT recipient presenting with back pain, as it has previously been described only in kidney transplant recipients [8]. Our patient experienced severe back pain with an intensity comparable to that reported for lower limb manifestation [4, 9, 19]. The unremarkable neurological and vascular examination, absence of vasomotor instability, and trophic skin changes ruled out neuropathy, vascular insufficiency, and RSDS. All laboratory exams were normal, except for an increased alkaline phosphatase level, like in other documented cases of CIPS. Although MRI performed in our patient showed no typical radiological signs, such as bone marrow edema, the diagnosis of CIPS was considered based on the clinical presentation and characteristics of the pain. Pain proved refractory to multiple analgesics, including opioids; only withdrawal of ciclosporin combined with pregabalin therapy produced a beneficial effect, suggesting the likely neuropathic mechanism of pain in CIPS [31]. This report has some limitations, including the lack of vitamin D assessment and the absence of radiological confirmation, which, however, is not consistently present in all reported cases of CIPS.

## Conclusion

Our case is an educational tool for transplant clinicians: musculoskeletal adverse effects of immunosuppressive therapy are rare and frequently misdiagnosed but should be considered in allo-HSCT recipients when more common causes have been ruled out and when investigations yield negative or non-significant results.

In the event of adverse reaction to a specific immunosuppressant, switching to another agent within the same class or to one acting on different pathways appears to be safe and beneficial.

## Data Availability

No datasets were generated or analysed during the current study.
